# Inclusive Leadership and Innovative Performance: A Multi-Level Mediation Model of Psychological Safety

**DOI:** 10.3389/fpsyg.2022.934831

**Published:** 2022-06-23

**Authors:** Tong Li, Ningyu Tang

**Affiliations:** Antai College of Economics and Management, Shanghai Jiao Tong University, Shanghai, China

**Keywords:** inclusive leadership, multi-level analysis, psychological safety, individual innovative performance, team innovative performance

## Abstract

Taking both individual and team levels into consideration has been called for years in terms of research on leadership. Inclusive leadership, a trending leadership style emerging from the global needs of managing the increasingly diversified workplace nowadays, has yet been rarely studied at both levels. To answer these calls, we specifically analyzed the relationship between inclusive leadership, team psychological safety, and innovative performance *via* a multilevel analysis. The results are based on a study of 356 employees from 90 working teams. Individual perceptions of inclusive leadership are positively related to the individual innovative performance through the mediation of individual psychological safety. Team perceptions of inclusive leadership are positively related to the team innovative performance through the mediation of team psychological safety. Moreover, team perceptions of inclusive leadership are positively related to the individual innovative performance through the cross-level mediation of individual psychological safety. Implications for both theory and practice are discussed.

## Introduction

Workforce diversity, catalyzed by economic globalization and technology development, has become a trend in the workplace (Pelled et al., 1999). The diversity not only includes demographic factors like gender, age, and living status but also involves underlying levels such as values and social cognition (Harrison et al., 1998). Therefore, the diversity of employees in the workplace brings complications for the management, such as serious problems of inequality and discrimination in the workplace (Kelly and Dobbin, 1998; Mor Barak et al., 2003; Mor Barak, 2007; Smith et al., 2012). To deal with the challenges brought by workforce diversity, organizational leaders have become increasingly aware of the importance of creating an inclusive environment (Nishii and Rich, 2014). Furthermore, inclusive leadership emerges as an ideal leadership style to motivate diversified employees to better realize themselves as well as to treat them fairly (Roberson, 2006). Through fair treatment of and providing strong motivation to the employees from diversified backgrounds, inclusive leadership may positively influence the performance at both individual and organizational levels (Pless and Maak, 2004; Echols, 2009; Shore et al., 2011; Bowers et al., 2012; Tang and Zhang, 2015).

Among the positive effects that inclusive leadership exerts on multiple kinds of individual or organizational performance, this study mainly focuses on the relationship between inclusive leadership and innovative performance for two reasons. First, innovative performance is recognized as crucial for organizational success and survival (Amabile, 1988), which makes it essential to explore ways to improve the innovative performance of organizations. In this study, we attempted to examine whether a specific style of leadership, inclusive leadership, would elevate innovative performance. Second, previous studies have proved that workforce diversity wields a positive influence on both employees' and organization's innovative performance (Govendo, 2005; Mohammadi et al., 2017). Thus, it is worth studying whether inclusive leadership, which is developed to manage workforce diversity, can also boost innovative performance.

Despite scholars' growing interests in inclusive leadership, previous studies on this topic mostly focus on the individual level, which indicates limited knowledge. This is mainly because the structure of organizations has been evolving from individual-based to team-based, and supervisors are more frequently requested to lead employees as individuals as well as teams (Cohen and Bailey, 1997; Hackman, 2002; Kozlowski and Bell, 2003). The team-based trend prompted a series of management issues from both theoretical and practical perspectives (Chen and Kanfer, 2006). Leading teams rather than individuals raises new questions, such as how team-focused leadership affects individuals, and whether trade-offs may occur between managing teams and individuals (Chen et al., 2007; Wang and Howell, 2012). Therefore, research on leadership should integrate individual- and team-level processes to answer these questions (Kozlowski and Bell, 2003).

To further fill the gap, the current study intends to examine the multilevel influences of inclusive leadership on innovative performance. Drawing from the social exchange theory (Blau, 1964), we argue that inclusive leadership offers employees higher levels of psychological safety, which is a kind of desirable psychological resource. This in turn draws employees to present better innovative performance as a return to their inclusive leaders. As inclusive leadership and team psychological safety are both team-level constructs and hold individual perceptions, we bring up our assertions according to the direct consensus model (Chan, 1998).

The current study makes three contributions to the existing research. First, this study reviewed a modest number of studies that explore the influences of leadership on the relevant outcomes at both individual and team levels. Second, we extended the previous research on the positive effect of inclusive leadership on innovative performance by examining how inclusive leadership promotes innovative performance at both levels and by cross-level means. Third, we expanded the literature on psychological safety in terms of the multilevel analysis by proving it to be a cross-level mediator within the relationship between inclusive leadership and innovative performance.

## Theoretical Backgrounds and Hypotheses

### Inclusive Leadership and Innovative Performance

In the current study, we developed an overall theory mainly from the perspective of the social exchange theory. According to the social exchange theory, when leaders provide material or nonmaterial resources to employees, they will form an emotional social exchange relationship with employees, which makes employees generate the willingness and take on action to give back to their leaders (Blau, 1964). Therefore, we argue that inclusive leadership, which allows employees to perceive belongingness and present uniqueness at the same time (Roberson, 2006), provides lots of psychological supportive resources to help employees live comfortably in organizations. In return, employees would repay their leaders by working harder and pursuing higher performance such as innovative performance.

As Yammarino and Dansereau (2008) argued, leadership is a multilevel construct in nature. Considering this inherent multilevel characteristic, as well as few studies on inclusive leadership that covered multilevel interplay, the current study investigated the influence of inclusive leadership on innovative performance at multiple levels. According to Chun et al. (2009), the multilevel construct should consider the difference between teams, the difference within teams, and the difference between followers independent of their teams. Therefore, we have discussed our research model from several plausible levels.

#### Individual-Level Relationships

Inclusive leadership, constructed as motivating employees' participation and achieving organizational support by showing three attributes, namely, openness, availability, and accessibility (Edmondson, 2004), is an important method to manage the workforce diversity and achieve organizational inclusion (Pless and Maak, 2004). Some earlier studies investigated the effects of inclusive leadership on innovative working behaviors at the individual level and provided evidence mostly for positive relations (Carmeli et al., 2010; Javed et al., 2018, 2019a). The perception of inclusive leadership may foster individual innovative performance in three aspects. First, according to the social exchange theory, when inclusive leaders are perceived to provide assistance and support to employees, employees would feel obliged to repay the leader and organization (Blau, 1964). Thus, employees are more likely to reciprocate by displaying extra-role behavior such as innovative working behaviors (Pless and Maak, 2004; Choi et al., 2015). Second, inclusive leaders are perceived to be open to employees expressing their views and to be always available and accessible to be their listeners, which guarantees that employees can freely generate and present new ideas without being ignored or rejected (Carmeli et al., 2010). Third, by actively communicating and providing assistance to employees, inclusive leaders make employees feel supported by their leaders (Javed et al., 2019b), which makes employees more likely to perform innovative behaviors (Clegg et al., 2002; Janssen, 2005). To sum up, we conclude that individual perceptions of inclusive leadership would encourage employees to express novel ideas as well as to transform the ideas into innovative behaviors and further improve their innovative performance. Hence, we hypothesize the following:

**Hypothesis 1**: Individual perceptions of inclusive leadership are positively related to individual innovative performance.

#### Team-Level Relationships

According to Braun et al. (2013, p. 271), “if theoretical constructs relate to individuals nested in teams, one must acknowledge the team as a meaningful entity.” Thus, in addition to considering individual perceptions of inclusive leadership, we should investigate the team perceptions of inclusive leadership as well.

Unlike individual performance, to improve team performance, leaders consider not only how to improve performance at the individual level but also how to display team-focused behaviors that promote shared commitment to teams and promote positive team processes (Kozlowski et al., 1996; Morgeson et al., 2010). Despite that we mentioned inclusive leadership may positively affect individual innovative performance, these individuals, as team members, need to be encouraged to commit to the team and cooperate as a whole to improve the team's innovative performance. As the characteristics of inclusive leadership (openness, availability, and accessibility) help leaders to focus on facilitating group members to feel themselves as part of the group (belongingness) and retaining their sense of individuality (uniqueness) while contributing to the group processes and outcomes, employees can all feel inclusiveness and further get committed to the team (Randel et al., 2018). This commitment to the team can also be considered as team members' giving back to their leaders' inclusiveness from the perspective of the social exchange theory (Blau, 1964). Moreover, by overcoming the barriers between team members from different backgrounds, inclusive leadership can build a positive social environment where team members are more aware of the team goals and increase work coordination (Wasserman et al., 2008; Mor Barak, 2013; Qi and Liu, 2017), which might be the most important mediating mechanism for team outcomes (Anderson and West, 1998). Overall, at the team level, inclusive leadership improves the commitment to teams of team members and shapes a comprehensive work atmosphere where team members feel comfortable to generate innovative ideas and cooperate with each other to accomplish team innovation. Thus, we hypothesize the following:

**Hypothesis 2**: Team perceptions of inclusive leadership are positively related to team innovative performance.

#### Cross-Level Relationships

Furthermore, we suggest that team perceptions of inclusive leadership exert a cross-level main effect on the individual innovative performance. In this case, the individual innovative performance will not only be improved by inclusive leadership experiencing indirect interactions with the supervisor but also by leadership behaviors that are directed toward other team members or the team as a whole. For one reason, inclusive leaders may accept new information, listen to a new voice, and receive a new challenge (Hirak et al., 2012), which encourages every team member to generate and express creative ideas. We believe that when someone in the team witnesses other members getting appreciated for exhibiting innovative behaviors by inclusive leaders, he or she may probably suppose that it is appropriate to act the same. Thus, inclusive leadership can affect individuals by affecting other team members. For another, by creating “an environment that acknowledges, welcomes, and accepts different approaches, styles, perspectives, and experiences” (Winters, 2014, p. 206), inclusive leadership effectively manages the workforce diversity and positively influences the team process by promoting coordination and mitigating conflicts (Qi and Liu, 2017; Randel et al., 2018). The environment created by inclusive leaders not only benefits the teams they lead but also makes every team member feel supported and energized to better engage in their tasks to repay their leaders (Pless and Maak, 2004). Hence, we further argue that individual innovative performance can be improved by these team-focused inclusive leading behaviors. We hypothesize the following:

**Hypothesis 3**: Team perceptions of inclusive leadership are positively related to individual innovative performance.

### Psychological Safety as a Mediator

Psychological safety is defined as perceptions of the consequences of taking interpersonal risks in a particular context such as a workplace (Edmondson, 1999). In this study, we introduced psychological safety as a mediator from the perspective of the social exchange theory (Blau, 1964). As we discussed before, the three characteristics, openness, availability, and accessibility of inclusive leadership, make employees feel supported and behave more comfortably in organizations (Edmondson, 2004), so they would put more effort into extra-role behavior such as improving innovative performance to repay their leaders' kindness. In this logic, we further put forth that psychological safety is one of the essential psychological resources that employees receive from an inclusive leadership style (Carmeli et al., 2010), which would further drive them to repay their leaders with higher innovative performance.

Though first brought up as a team-level construct (Edmondson, 1996, 1999), research on psychological safety has gained fruitful findings by treating psychological safety as a phenomenon at multiple levels including individual level, group/team level, and organization level (Edmondson and Lei, 2014). Edmondson and Lei (2014) also mentioned that, despite the multilevel findings, research on psychological safety should pay attention to how phenomena at different levels of analysis interact. Thus, we separately discuss the mediating role of psychological safety at different levels in this section.

#### Individual-Level Mediation

We first argue that individual perceptions of inclusive leadership will positively influence individual psychological safety. According to the essence of inclusive leadership, if an individual perceives a leader to be inclusive, he/she is likely to consider the leader to be open, available, and accessible in the leader–follower relationship. As Edmondson (2004) addressed, the openness, availability, and accessibility exhibited by leaders are likely to promote the development of psychological safety. By being open, inclusive leaders actively communicate with employees about achieving work goals or catching new opportunities, and the feeling of being invited and appreciated for their voice would help facilitate the development of employees' psychological safety (Nembhard and Edmondson, 2006). By being available and accessible, inclusive leaders send a clear signal that employees can easily get in touch with them and address issues (Carmeli et al., 2010). Therefore, when individuals perceive high levels of leader inclusiveness, they would feel safe to reach out to the leader and express their ideas without worrying about causing interpersonal risk, which assists employees to develop individual psychological safety.

Then, we propose that individual psychological safety fosters individual innovative performance. To achieve higher innovative performance, an employee needs to generate creative ideas and exhibit innovative behaviors. However, employees may take risks in the context of innovation by proposing and implementing new ideas, since many of them could end up with organizational failure (Janssen, 2002; Mathisen et al., 2012; Javed et al., 2019b). Therefore, employees need support in terms of psychological safety to alleviate the risks and become involved in the innovation process and realize their creative potential (Harrington et al., 1987). Indeed, Kark and Carmeli (2009) proved that psychological safety induces feelings of vitality to impact an individual's involvement in creative work. Carmeli et al. (2010) further proved that psychological safety positively influences employees' involvement in creative behaviors, which is important for their creative performance. Therefore, we suggest that, at the individual level, higher psychological safety would motivate employees to create more original ideas, get more involved in innovative behaviors, and eventually improve their innovative performance.

To conclude, we discuss that perceptions of inclusive leadership would make individuals feel safe to voice their feelings and perform and develop feelings of psychological safety, which may sequentially drive individuals to engage in innovative work and manifest higher innovative performance. We hypothesize the following:

**Hypothesis 4**: Individual psychological safety mediates the positive relationship between individual perceptions of inclusive leadership and individual innovative performance.

#### Team-Level Mediation

The analysis of team psychological safety originated from the research by Edmondson (1996, 1999), in which she posited team psychological safety as a shared belief by team members that the team is safe for interpersonal risk-taking. In a similar vein, we first discuss the relationship between team perceptions of inclusive leadership and team psychological safety. Team perceptions of inclusive leadership imply the average level at which the leader is perceived to be inclusive by the team. If a leader is perceived to be inclusive in the context of a team, he/she may be perceived to acknowledge and respect the uniqueness of team members, invite and listen to team members to express their suggestions and concerns, and motivate team members to implement ideas without worrying about the risk of being criticized and punished (Ye et al., 2019). Moreover, inclusive leaders may exhibit socio-emotional support behaviors to develop strong emotional links and interpersonal relationships with team members (Hollander, 2009). Thus, inclusive leadership would cause team members to feel safe to take interpersonal risks in the team, which, in other words, facilitates feelings of psychological safety of team members. Furthermore, we suggest that the feelings of safety of every team member would converge into a shared belief as team psychological safety.

Then, we conceive that team psychological safety relates positively to team innovative performance in several ways. As we mentioned earlier, team innovative performance refers to more than a simple aggregation of individual innovative performance. Team members need to cooperate with each other and work as a whole to promote team innovation. Indeed, psychological safety has been identified as an essential factor in understanding how people in a group collaborate to achieve a shared outcome in organizational research (Edmondson, 1999, 2004). In specific, in a team with high psychological safety, team members are found to be more likely to question suggestions and decisions (Burke et al., 2006), share knowledge (Collins and Smith, 2006; Siemsen et al., 2009), and join team learning (Huang et al., 2008). Moreover, through these team processes, team members would take more initiatives to develop new products and services (Baer and Frese, 2003), which can lead to a better team innovative performance. Altogether, we argue that team perceptions of inclusive leadership would nourish a shared belief of team psychological safety, which will further promote team cooperation and improve team innovative performance. We conclude and suggest the following hypothesis:

**Hypothesis 5**: Team psychological safety mediates the positive relationship between team perceptions of inclusive leadership and team innovative performance.

#### Cross-Level Mediation

Finally, we propose that the cross-level relationship between the team perceptions of inclusive leadership and individual innovative performance is mediated by individual psychological safety. So far, we have discussed how individual psychological safety encourages individuals to perform better at innovative work, but we need to further investigate how a team's perceptions of inclusive leadership cultivate individual psychological safety. That is, individual psychological safety would be improved by not only the inclusive leadership experienced in indirect interactions with the leader but also the leader's behaviors toward other team members or the team as a whole. For one thing, inclusive leaders are open to opinions and suggestions generated by team members and always ready to get reached by team members for communication and discussion (Carmeli et al., 2010). By doing this, inclusive leaders ensure that every team member feels psychologically safe and takes no worry about interpersonal risks. We believe that, when team perceptions of inclusive leadership are high, even if an individual does not receive the inclusiveness by oneself, he/she would probably witness other team members being included to take actions in teams without worrying about being criticized or punished. As a result, he/she will develop a sense of psychological safety because he/she can act just like everyone else and get treated equally by the inclusive leaders. For another thing, inclusive leadership influences the team as a whole by motivating team processes such as knowledge sharing and team learning (Huang et al., 2008; Siemsen et al., 2009). Though directed toward the whole team, these processes can make every team member benefit. With more interaction and cooperation with other team members, an individual may develop stronger bonds with the others, which may trigger a higher level of psychological safety (Edmondson and Mogelof, 2005). Moreover, inclusive leadership shapes and maintains a favorable work environment and cultural norm in teams (Carmeli et al., 2010), in which every team member would be impacted by the safe and comfortable climate and feel psychologically safe in the team. Combining with earlier discussion, we conclude that a team-level inclusive leadership would have a cross-level influence on individual psychological safety, which would in turn promote better individual innovative performance. We hypothesize the following:

**Hypothesis 6**: Individual psychological safety mediates the positive relationship between the team's perceptions of inclusive leadership and individual innovative performance.

**Figure 1:**
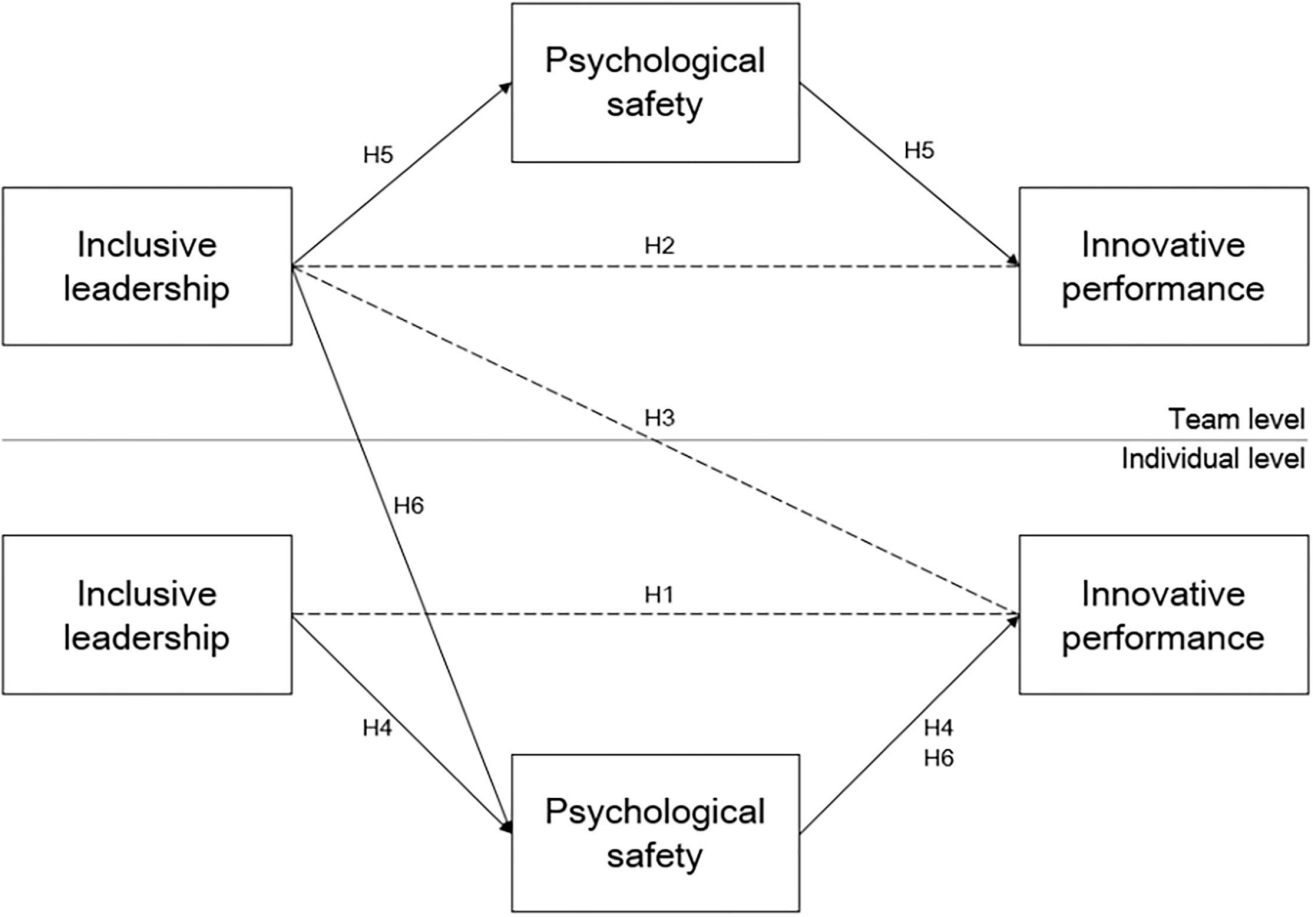
Research model.

In conclusion, Figure 1 presents the multilevel mediation model of inclusive leadership, psychological safety, and innovative performance examined in this study.

## Methodology

### Participants and Procedure

We collected data for this study from the operating departments of two large companies (one from the automobile industry and another one from the chemical industry, respectively) located in northern and southern China. With the help of the HR department, we were able to distribute surveys to participants during their work hours on site. Due to the risk of lockdown policy during the COVID-19 pandemic, we collected the data in one wave. To prevent the common method bias that may be caused by one-wave data collection, we employed a paired-questionnaire design. The survey consists of two versions. The supervisor version, rated by the team leaders, includes measurements of the individual innovative performance, team innovative performance, and team-level controls. The employee version is rated by employees and composed of measurements on inclusive leadership, psychological safety, and individual-level controls. We invited 102 working teams to take part in the study and distributed paper questionnaires to both team leaders and team members, and 12 team leaders did not respond to the surveys. Hence, the final sample for this study consists of 90 teams (including 90 questionnaires from team leaders and 356 questionnaires from team members, representing response rates of 88.2%).

Within the sample of 90 team leaders, 86.7% are men, 72.2% are married, 51.1% grew up in rural areas, and their average age is 33.7 years old. In terms of educational level, 28.9% have a high school degree and below, 15.6% have a college degree, 45.6% have a bachelor's degree, and 10% have a master's degree and above. Team leaders' job tenure ranged from <3 years (8.9%), 3–5 years (20.0%), 5–8 years (15.6%), 8–10 years (11.1%), and more than 10 years (44.4%). Team leaders' job positions ranged from general staff (6.7%), junior manager (53.3%), middle-level manager (35.6%), and senior manager (4.4%).

Within the sample of team members, 72.2% are men, 43.0% are married, 52.5% grew up in rural areas, and their average age is 28.2 years old. In terms of educational level, 32.3% have a high school degree and below, 22.5% have a college degree, 39.0% have a bachelor's degree, and 6.2% have a master's degree and above. Team members' job tenure ranged from <3 years (33.7%), 3–5 years (28.4%), 5–8 years (11.5%), 8–10 years (6.7%), and more than 10 years (19.7%). Team members' job position ranged from general staff (92.4%), junior manager (7.0%), middle-level manager (0.6%), and senior manager (0%).

### Measures

Although the original measurements were in English, our study was conducted in the Chinese context, so we followed the back-translation procedures recommended by Brislin (1970) for survey translation across different languages. All measurements have a 5-point Likert-scale (1 = strongly disagree, 5 = strongly agree).

#### Inclusive Leadership

We used the nine-item scale adapted from Carmeli et al. (2010) to measure inclusive leadership. Sample items included the following: “The team leader is open to hearing new ideas;” “The team leader is available for consultation on problems;” and “The team leader encourages me to access him/her on emerging issues.” The Cronbach's α for the scale in our study is 0.942.

#### Psychological Safety

We use seven items adapted from Edmondson (1999) to measure psychological safety. Sample items included the following: “If I make a mistake on this team, it is often held against me” (Reverse) and “It is safe to take a risk on this team.” The Cronbach's α for the scale in our study is 0.741.

#### Individual Innovative Performance

We measure the individual innovative performance by adapting four items of the “innovator role” from Welbourne et al.'s role-based performance scales (Welbourne et al., 1998). Sample items included the following: “The employee does a good job at coming up with new ideas” and “The employee does well in working to implement new ideas.” The Cronbach's α for the scale in our study is 0.944.

#### Team Innovative Performance

We used a four-item scale adapted from Lovelace et al. (2001) to measure the team's innovative performance. Sample items included the following: “The innovativeness of the team's product is good” and “The number of innovations or new ideas introduced by the teams is outstanding.” The Cronbach's α for the scale in our study is 0.710.

#### Control Variables

We controlled for possible alternative explanations by including both individual-level and team-level control variables. Previous studies suggested some demographic variables of both team leader and team members for controlling, such as gender (1 = male, and 2 = female), age (self-reported in numbers), educational level (1 = high school degree and below, 2 = college, 3 = bachelor's degree, and 4 = master's degree and above), marital status (1 = married, and 2 = not married), birthplace (1 = rural and 2 = urban), job tenure (1 = less than 3 years, 2 = 3–5 years, 3 = 5–8 years, 4 = 8–10 years, and 5 = more than 10 years), and job position (1 = general staff, 2 = junior manager, 3 = middle-level manager, and 4 = senior manager; Carmeli et al., 2010; Javed et al., 2018). Besides, we controlled for team size at the team level according to previous findings that team size is related to both psychological safety and innovative performance (Hülsheger et al., 2009; Edmondson and Lei, 2014; Ye et al., 2019).

The summary of all the constructs employed in this study is presented in Table 1.

**Table 1:** Summary of constructs in this study.

**Construct**	**References**	**Sample item**	**Cronbach's α**
Inclusive leadership	Carmeli et al. (2010)	The team leader is open to hearing new ideas.	0.942
Psychological safety	Edmondson (1999)	It is safe to take a risk on this team.	0.741
Individual innovative performance	Welbourne et al. (1998)	The employee does a good job at coming up with new ideas.	0.944
Team innovative performance	Lovelace et al. (2001)	The innovativeness of the team's product is good.	0.710

### Analytic Strategy

We employed the software AMOS 23.0, SPSS 25.0, and HLM 7.0 to test our proposed research model. First, we conducted a confirmatory factor analysis (CFA) with AMOS 23.0 to assess the discriminant validity of core variables in the model. Second, correlation analysis was conducted to assess the correlations of the variables in the model. Third, we conducted hierarchical regression analysis with the SPSS 25.0 to test hypotheses at the mono-level. Last, we conducted hierarchical linear modeling with HLM 7.0 to test the cross-level hypotheses. The hierarchical linear modeling considered both individual- and team-level residuals, which can recognize the partial interdependence among individuals in the same team. We modeled inclusive leadership as a team-level variable, and to support the aggregation of team members' perceived inclusive leadership to the team-level variable, we also calculated within-team agreement, intraclass correlations, and reliabilities of the means.

## Results

### Common Method Bias Test

Though our data was collected from different sources (team leaders and team members), they were also measured at the same time, which introduced the potential for common method variance. For this reason, we employed the widely used Harman's single-factor test. The results of the test showed that the first factor explained only 31.506% of the variance, which is lower than half of the total variance explained (64.679%) as well as the critical value of 40%. Because of the limitations of this method, we also examined the effects of adding a latent common methods factor to the hypothesized measurement model (Podsakoff et al., 2003). The fit of this model is not significantly better than that of the hypothesized model (Δχ^2^ = 10.662, Δ*Df* = 9). Meanwhile, the variance extracted by the common methods factor was only 0.185, falling below the 0.500 cutoff that has been suggested as indicating the presence of a latent factor representing the manifest indicators (Hair et al., 1998). Therefore, both results suggest that common method bias has been sufficiently controlled in our study.

### Validity Analysis

#### Data Aggregation

As inclusive leadership and psychological safety at the team level refer to the shared perceptions of the team members, we aggregated the individual perceptions of these two variables to yield the measures at the team level. To assess the appropriateness of aggregation, we calculated within-team agreement (Rwg; James et al., 1984), intraclass correlations (ICC1), and reliabilities of the means (ICC2; Bliese, 2000). The Rwg, ICC1, and ICC2 values supported aggregating individual scores to the team level of analysis (inclusive leadership: ICC1 = 0.475, ICC2 = 0.782, Rwg = 0.972; psychological safety: ICC1 = 0.378, ICC2 = 0.706, Rwg = 0.964; James, 1982; James et al., 1993).

#### Construct Validation

Before examining our hypotheses, we performed a CFA to evaluate the construct distinctiveness of the measurement model consisting of perceived inclusive leadership, perceived psychological safety, and individual innovative performance. We used item parceling based on the single-factor method (Bentler and Chou, 1987) to check if all the measurement items are included as observed indicators (Landis et al., 2000). We constructed an individual-level model with three factors, loading separately. Table 2 presents the results of CFA, indicating that the hypothesized three-factor model (χ^2^ = 32.344, *Df* = 24, χ^2^/*Df* = 1.348 TLI = 0.992, CFI = 0.995, RMSEA = 0.031) fits better than the other competitive models.

**Table 2:** Results of confirmatory factor analysis.

**Model**	**Factors**	**χ^2^**	**Df**	**χ^2^/Df**	**CFI**	**TLI**	**RMSEA**
Three-factor model	PIL, PPS, IIP	32.344	24	1.348	0.995	0.992	0.031
Two-factor model 1	PIL+PPS, IIP	132.954	26	5.114	0.933	0.908	0.108
Two-factor model 2	PIL, PPS+IIP	188.042	26	7.232	0.899	0.860	0.132
Single-factor model	PIL+PPS+IIP	661.515	27	24.501	0.605	0.473	0.257

*PIL, Individual perceptions of inclusive leadership; PPS, Individual Perceptions of psychological safety; IIP, Individual innovative performance*.

### Descriptive Statistics

Table 3 shows the means, standard deviations, and correlations of key variables at the individual level. The results indicate that individual perceptions of inclusive leadership are positively correlated with the individual perceptions of psychological safety (*r* = 0.434, *p* < 0.01) and individual innovative performance (*r* = 0.295, *p* < 0.01). The individual perceptions of psychological safety are positively correlated with \ individual innovative performance (*r* = 0.323, *p* < 0.01).

**Table 3:** Means, standard deviations, and correlations among variables at the individual level.

**Variable**	**M**	**S.D**.	**1**	**2**	**3**	**4**	**5**	**6**	**7**	**8**	**9**
1. Team member's gender	1.278	0.449									
2. Team member's age	28.219	6.118	0.184^**^								
3. Team member's educational level	2.191	0.963	0.085	−0.170^**^							
4. Team member's marital status	1.570	0.496	−0.132^*^	−0.590^**^	0.161^**^						
5. Team member's birthplace	1.475	0.500	0.126^*^	0.212^**^	0.033	−0.106^*^					
6. Team member's job tenure	2.503	1.498	0.273^**^	0.743^**^	−0.248^**^	−0.641^**^	0.166^**^				
7. Team member's job position	1.081	0.294	0.063	0.106^*^	0.194^**^	−0.146^**^	0.062	0.163^**^			
8. Perceived inclusive leadership	3.902	0.537	0.066	0.026	0.077	−0.042	−0.089	0.019	0.005		
9. Perceived psychological safety	3.453	0.377	0.063	−0.038	0.094	0.071	0.048	−0.021	0.000	0.434^**^	
10. Individual innovative performance	4.276	0.864	−0.033	0.017	0.056	−0.030	−0.107^*^	0.013	−0.003	0.295^**^	0.323^**^

*n = 356*.

** *p < 0.01*,

* *p < 0.05, reliabilities are mentioned in parentheses on the diagonal*.

Table 4 shows the means, standard deviations, and correlations of key measured variables at the team level. The results indicate that team perceptions of inclusive leadership are positively correlated with team psychological safety (*r* = 0.538, *p* < 0.01) and team innovative performance (*r* = 0.367, *p* < 0.01). Team psychological safety is positively correlated with team innovative performance (*r* = 0.360, *p* < 0.01).

**Table 4:** Means, standard deviations, and correlations among variables at the team level.

**Variable**	**M**	**S.D**.	**1**	**2**	**3**	**4**	**5**	**6**	**7**	**8**	**9**	**10**
1. Team leader's gender	1.133	0.342										
2. Team leader's age	33.733	8.829	0.053									
3. Team leader's educational level	2.367	1.011	0.020	0.301^**^								
4. Team leader's marital status	1.278	0.450	−0.097	−0.512^**^	−0.004							
5. Team leader's birthplace	1.489	0.503	0.139	0.017	−0.003	0.039						
6. Team leader's job tenure	3.622	1.442	0.081	0.753^**^	0.173	−0.650^**^	−0.068					
7. Team leader's job position	2.378	0.680	0.023	0.335^**^	0.646^**^	−0.016	−0.020	0.227^*^				
8. Team size	3.956	1.005	0.148	0.132	0.072	−0.097	0.088	0.027	0.140			
9. Inclusive leadership	3.907	0.424	0.092	0.324^**^	−0.087	−0.252^*^	−0.015	0.445^**^	−0.140	−0.041		
10. Team psychological safety	3.448	0.280	0.121	0.301^**^	0.073	−0.130	0.109	0.318^**^	−0.129	0.066	0.538^**^	
11. Individual innovation performance	3.625	0.486	−0.186	0.251^*^	0.163	−0.212^*^	−0.149	0.256^*^	0.068	0.069	0.367^**^	0.360^**^

*n = 90*.

** *p < 0.01*,

* *p < 0.05, reliabilities are mentioned in parentheses on the diagonal*.

### Hypotheses Testing

#### Hypotheses Testing at the Individual Level

We conducted a hierarchical regression analysis at the individual level to test the impact of individual perceptions of inclusive leadership on individual innovative performance and the mediating role of individual perceptions of psychological safety between the two variables. Table 5 presents the results of the hierarchical regression analyses at the individual level.

**Table 5:** Results of hypotheses testing at the individual level.

**Variables**	**Individual perceptions of psychological safety**		**Individual innovative performance**			
	**M1**	**M2**	**M3**	**M4**	**M5**	**M6**
Team member's gender	0.051	0.023	−0.041	−0.060	−0.058	−0.065
Team member's age	−0.040	−0.055	0.025	0.015	0.038	0.030
Team member's educational level	0.087	0.048	0.084	0.059	0.055	0.046
Team member's marital status	0.085	0.107^+^	−0.030	−0.016	−0.058	−0.043
Team member's birthplace	0.046	0.095^+^	−0.117^**^	−0.086	−0.132^**^	−0.110^**^
Team member's job tenure	0.066	0.071	0.030	0.034	0.008	0.015
Team member's job position	−0.017	−0.009	−0.021	−0.016	−0.016	−0.014
Individual perceptions of inclusive leadership		0.442^***^		0.285^***^		0.171^**^
Individual perceptions of psychological safety					0.333^***^	0.258^***^
**Index**						
*F*	1.001	11.560^***^	1.030	4.799^***^	6.441^***^	6.905^***^
*R* ^2^	0.020	0.210	0.020	0.100	0.129	0.152
Δ*R*^2^	–	0.191	–	0.079	0.109	0.053

+ *p < 0.1*,

* *p < 0.05*,

** *p < 0.01*,

*** *p < 0.001*.

As can be seen from model 4, individual perceptions of inclusive leadership are significantly related to individual innovative performance (β = 0.285, *p* < 0.001). Thus, hypothesis 1 is supported. In model 2, the individual perceptions of inclusive leadership are significantly related to individual perceptions of psychological safety (β = 0.442, *p* < 0.001). After entering individual perceptions of psychological safety as the mediator in model 6, individual perceptions of psychological safety are positively related to individual innovative performance (β = 0.258, *p* < 0.001), and individual perceptions of inclusive leadership are still significantly related to individual innovative performance (β = 0.171, *p* < 0.01) but weaker than that in model 4. Therefore, the results reveal a significant mediating effect of the individual perceptions of psychological safety on the relationship between the individual perceptions of inclusive leadership and individual innovative performance (Baron and Kenny, 1986; Wu, 2008). Thus, hypothesis 4 is supported.

#### Hypotheses Testing at the Team Level

Similarly, we conducted hierarchical regression analyses at the team level to test the influence of team perceptions of inclusive leadership on team innovative performance and the mediating role of team psychological safety. Table 6 presents the results of the hierarchical regression analyses at the team level.

**Table 6:** Results of hypotheses testing at the team level.

**Variables**	**Team psychological safety**		**Team innovative performance**			
	**M1**	**M2**	**M3**	**M4**	**M5**	**M6**
Team leader's gender	0.083	0.052	−0.207^*^	−0.234^*^	−0.238^*^	−0.247^*^
Team leader's age	0.194	0.149	0.095	0.056	0.023	0.018
Team leader's educational level	0.222^+^	0.237^*^	0.192	0.206	0.110	0.145
Team leader's marital status	0.213	0.153	−0.086	−0.138	−0.165	−0.177
Team leader's birthplace	0.096	0.096	−0.120	−0.120	−0.156	−0.145
Team leader's job tenure	0.367^*^	0.137	0.131	−0.066	−0.005	−0.101
Team leader's job position	−0.428^**^	−0.310^*^	−0.130	−0.029	0.029	0.051
Team size	0.075	0.086	0.090	0.100	0.063	0.078
Team perceptions of Inclusive leadership		0.445^***^		0.381^***^		0.267^*^
Team psychological safety					0.371^***^	0.257^*^
**Index**						
*F*	3.275^**^	5.623^***^	1.943^+^	3.223^**^	3.203^**^	3.493^***^
*R* ^2^	0.244	0.387	0.161	0.266	0.265	0.307
Δ*R*^2^	–	0.143	–	0.105	0.104	0.040

+ *p < 0.1*,

* *p < 0.05*,

** *p < 0.01*,

*** *p < 0.001*.

As can be seen from model 4, team perceptions of inclusive leadership are significantly related to team innovative performance (β = 0.381, *p* < 0.001). Thus, hypothesis 2 is supported. In model 2, team perceptions of inclusive leadership are significantly related to team psychological safety (β = 0.445, *p* < 0.001). After entering team psychological safety as the mediator in model 6, team psychological safety is positively related to team innovative performance (β = 0.257, *p* < 0.05), and team perceptions of inclusive leadership are still significantly related to team innovative performance (β = 0.267, *p* < 0.05) but weaker than that in model 4. Therefore, the results revealed a significant mediating effect of team psychological safety on the relationship between team perceptions of inclusive leadership and team innovative performance (Baron and Kenny, 1986; Wu, 2008). Thus, hypothesis 5 is supported.

#### Hypotheses Testing of the Cross-Level Effect

Table 7 presents the HLM results of testing the cross-level effect of the team's perceptions of inclusive leadership on individual innovative performance through individual perceptions of psychological safety. First, we examined whether there is a significant between-team variance in innovative performance. We followed the procedure recommended by Bryk and Raudenbush (1992) and conducted a null hierarchical linear modeling (HLM) analysis. The results revealed that the interclass correlation coefficient (ICC1) of individual innovation performance was 0.285/(0.285+0.475)=0.375>0.060. This finding provided a basis for examining individual-level and team-level predictors of individual innovative performance. Second, the results in model 2 revealed that team perceptions of inclusive leadership significantly helped to predict individual innovative performance (γ_01_ = 0.608, *p* < 0.001). Therefore, hypothesis 3 is supported. Third, the HLM results in model 3 revealed that team perceptions of inclusive leadership were significantly correlated with individual perceptions of psychological safety (γ_01_ = 0.360, *p* < 0.001). After we put both the independent variable and the mediator into model 4, the HLM results showed that individual perceptions of psychological safety had a positive within-level impact (*u*_0_ = 0.512, *p* < 0.001) as well as a between-level impact (γ^02^ = 0.657, *p* < 0.05) on individual innovative performance. Meanwhile, team perceptions of inclusive leadership were still significantly correlated to individual innovative performance (γ_01_ = 0.368, *p* < 0.01) but weaker than before. Thus, perceived psychological safety partially mediated the relationship, which provides support for hypothesis 6.

**Table 7:** Results of hierarchical linear model.

**Model**	**Parameter estimation**					
	***γ_00_***	***γ_01_***	***γ_02_***	***u_**0**_***	**σ^2^**	**τ*_**00**_***
**M1: null model**						
L1: *IIP_*ij*_*=*β_0*j*_*+*r_*ij*_*	4.278^***^				0.475	0.285^***^
L2: *β_0*j*_*=*γ_00_*+*u_0*j*_*						
**M2: Team perceptions of inclusive leadership** ** → individual innovative performance**						
L1: *IIP_*ij*_*=*β_0*j*_*+*r_*ij*_*	1.903^***^	0.608^***^			0.476	0.222^***^
L2: *β_0*j*_*=*γ_00_*+γ_01_*(*TIL_*j*_*) +*u_0*j*_*						
**M3: Team perceptions of inclusive leadership** ** → individual perceptions of psychological safety**						
L1: *IPS_*ij*_*=*β_0*j*_*+*r_*ij*_*	2.046^***^	0.360^***^			0.089	0.030^***^
L2: *β_0*j*_*=*γ_00_*+γ_01_*(*TIL_*j*_*) +*u_0*j*_*						
**M4: inclusive leadership** ** → perceived psychological safety** ** → individual innovation performance**						
L1: *IIP_*ij*_*=*β_0*j*_*+β_1j_*(*IPS_*ij*_*) +*r_*ij*_*	0.572	0.368^**^	0.657^*^	0.512^***^	0.452	0.209^***^
L2: *β_0*j*_*=*γ_00_*+γ_01_*(*TIL_*j*_*) +γ_02_*(*TPS_*j*_*) +*u_0*j*_*						
L2: *β_1*j*_*=*γ_10_*+*u_1*j*_*						

* *p < 0.05*,

** *p < 0.01*,

*** *p < 0.001*.

*σ^2^ is the residual of level 1; τ_00_ is the residual of intercept, that is u_0j_*.

*IIP, Individual innovative performance; TIL, team perceptions of inclusive leadership; IPS, individual perceptions of psychological safety; TPS, team perceptions of psychological safety. In all models, level 1 variables are group-mean centered*.

In addition, we conducted a Sobel test to further examine the mediating effects (Sobel, 1982). The Sobel test's *z*-value of individual perceptions of psychological safety was 2.218, which exceeded the benchmark of 1.960, which further confirms the cross-level mediating effect.

## Discussion

### Theoretical Contribution

We believe that our study advances the previous research in three ways. First, we addressed the call to pay more attention to the multilevel nature of leadership (Bliese et al., 2002). As several previous studies only focused on the multilevel influences of transformational leadership (Wang and Howell, 2012; Braun et al., 2013) and authentic leadership (Braun and Nieberle, 2017), we further added inclusive leadership to our analysis to fill the gap. By examining the relationship between inclusive leadership and innovative performance at multiple levels, we responded to the suggestions from past studies that leadership research should incorporate individual and team relations (Schriesheim et al., 2006).

Second, we substantiated the theoretical and empirical findings on the positive influence of inclusive leadership on innovative performance. In addition to verifying the relationship at the individual level and team level separately as earlier studies did, we further analyze the cross-level influence of inclusive leadership on the individual innovative performance. The results prove that the effect of inclusive leadership can be exerted to motivate employees to perform better at team-level, individual-level, and cross-level, which enriches the knowledge of the multilevel positive influences of inclusive leadership.

Third, we extend the literature on the multilevel effects of psychological safety. Considering that psychological safety can be conceptualized as a phenomenon at different levels (Edmondson and Lei, 2014), we empirically examine that the team psychological safety mediates the relationship between team perceptions of inclusive leadership and team innovative performance (team-level mediation), individual perceptions of psychological safety mediate the relationship between individual perceptions of inclusive leadership and the individual innovative performance (individual-level mediation), and individual perceptions of psychological safety mediate the relationship between team perceptions of inclusive leadership and the individual innovative performance (cross-level mediation). The findings contribute to the understanding of the multilevel effects on psychological safety.

### Practical Implications

In addition to the theoretical contribution, our study provides several practical implications as well. To begin with, we remind the supervisors in organizations of the importance of inclusive leadership in motivating employees' innovative performance. Based on our multilevel findings, we suggest supervisors exhibit more inclusive behaviors because inclusive leadership is proven to exert a positive influence on employees' innovative performance from both the team level and the individual level. Furthermore, supervisors should not only facilitate the belongingness and respect the uniqueness of every individual but also advocate an inclusive environment and encourage team cooperation for teams as a whole.

Then, for organizations, inclusive leadership should be taken into account in the process of hiring, promoting, and training supervisors. As demonstrated in the present study, inclusive leadership, characterized by a leader's openness, availability, and accessibility, can enhance employees' psychological safety and sequentially improve their innovative performance. As promoting innovation is imperative for organizations in the current competitive environment (Chowhan et al., 2017; Hu et al., 2018), it is beneficial for organizations to appoint supervisors with high levels of inclusiveness.

Moreover, our study highlights the influence of psychological safety. With the growth of knowledge economies and teamwork presence, psychological safety has become an essential topic (Edmondson and Lei, 2014). According to the results of our study that higher psychological safety fosters better innovative performance of employees, we recommend supervisors to pay attention to establishing the psychological safety of employees. Furthermore, supervisors should notice that psychological safety might be affected by both team-focused and individual-focused leader behaviors as our study indicates. Hence, supervisors should work on facilitating employees' individual psychological safety as well as building a psychologically safe climate within the teams.

### Limitations and Future Directions

Despite the theoretical and practical implications presented by our study, there are also several issues worth noticing. First, due to the COVID-19 pandemic, the data were cross-sectional through the one-way collection, which made it difficult to infer a causal relationship between inclusive leadership and innovative performance *via* psychological safety in our study. Even though the causal effect can be supported by our theoretical discussion and findings from previous studies, future research should choose a multi-wave data collection procedure or longitudinal design to further improve the explaining power of the causal relationship.

Second, we exerted the measurements developed by Carmeli et al. (2010) in the Western context to examine the positive influence of inclusive leadership in the Chinese context. Although we carefully checked the reliability and validity of the scales in our study, the results may still have some deviation, which could potentially weaken the applicability of the results of our study. As Tang et al. (2015) addressed, inclusion and inclusive management in China may contain special contents. Thus, future research is suggested to develop new measures in the Chinese context and conduct more indigenous studies.

Third, we did not investigate the boundary conditions of the multilevel mediation model. Indeed, the mediation of psychological safety can be influenced by many other factors. For example, contextual factors such as organizational context and country or culture context, team characteristics, and other individual characteristics, such as job duties, could potentially affect the mediating role of psychological safety (Edmondson and Lei, 2014). Combined with the multilevel model of our study, future research should explore the boundary conditions at different levels to generate more inspiring findings. Besides, when investigating the boundary conditions, we also call for studies focused on more multilevel mediating mechanisms underlying the relationship between inclusive leadership and innovative performance, through which we can gain more knowledge about the positive influences of inclusive leadership.

## Data Availability Statement

The raw data supporting the conclusions of this article will be made available by the authors, without undue reservation.

## Ethics Statement

Ethical review and approval was not required for the study on human participants in accordance with the local legislation and institutional requirements. Written informed consent for participation was not required for this study in accordance with the national legislation and the institutional requirements.

## Author Contributions

Both authors listed have made a substantial, direct, and intellectual contribution to the work and approved it for publication.

## Funding

This research was supported by the National Natural Science Foundation of China (71672114).

## Conflict of Interest

The authors declare that the research was conducted in the absence of any commercial or financial relationships that could be construed as a potential conflict of interest.

## Publisher's Note

All claims expressed in this article are solely those of the authors and do not necessarily represent those of their affiliated organizations, or those of the publisher, the editors and the reviewers. Any product that may be evaluated in this article, or claim that may be made by its manufacturer, is not guaranteed or endorsed by the publisher.

## References

[B1] Amabile T. M. (1988). A model of creativity and innovation in organizations, in Research in Organizational Behavior, eds Staw B. M. Cummings L. L. (Stamford, CT: JAI Press), 123–167.

[B2] Anderson N. R. West M. A. (1998). Measuring climate for work group innovation: development and validation of the team climate inventory. J. Org. Behav. 19, 235–258. 10.1002/(SICI)1099-1379(199805)19:3<235::AID-JOB837>3.0.CO;2-C

[B3] Baer M. Frese M. (2003). Innovation is not enough: climates for initiative and psychological safety, process innovations, and firm performance. J. Org. Behav. 24, 45–68. 10.1002/job.179

[B4] Baron R. M. Kenny D. A. (1986). The moderator-mediator variable distinction in social psychological research: conceptual, strategic and statistical considerations. J. Personal. Soc. Psychol. 51, 1173–1182. 10.1037/0022-3514.51.6.1173 3806354

[B5] Bentler P. M. Chou C. P. (1987). Practical issues in structural modeling. Sociol. Methods Res. 16, 78–117. 10.1177/0049124187016001004

[B6] Blau P. M. (1964). Exchange and Power in Social Life. Piscataway, NJ: Transaction Publishers.

[B7] Bliese P. D. (2000). Within-group agreement, non-independence, and reliability: implications for data aggregation and analysis, in Multilevel Theory, Research, and Methods in Organizations: Foundations, Extensions, and New Directions, eds Klein K. J. Kozlowski S. W. J. (Hoboken, NJ: Jossey-Bass), 349–381.

[B8] Bliese P. D. Halverson R. R. Schriesheim C. A. (2002). Benchmarking multilevel methods in leadership: the articles, the model, and the data set. Leadersh. Quart. 13, 3–14. 10.1016/S1048-9843(01)00101-1

[B9] Bowers K. W. Robertson M. Parchman M. L. (2012). How inclusive leadership can help your practice adapt to change: the most effective leaders realize that everyone's input is valuable. Family Practice Manag. 19, 8–11. PMC3796957 22335375

[B10] Braun S. Nieberle K. W. A. M. (2017). Authentic leadership extends beyond work: a multilevel model of work-family conflict and enrichment. Leadersh. Quart. 28, 780–797. 10.1016/j.leaqua.2017.04.003

[B11] Braun S. Peus C. Weisweiler S. Frey D. (2013). Transformational leadership, job satisfaction, and team performance: a multilevel mediation model of trust. Leadersh. Quart. 24, 270–283. 10.1016/j.leaqua.2012.11.006

[B12] Brislin R. W. (1970). Back-translation for cross-cultural research. J. Cross Cult. Psychol. 1, 185–216. 10.1177/135910457000100301

[B13] Bryk A. S. Raudenbush R. W. (1992). Hierarchical Linear Models: Applications and Data Analysis Methods. Newcastle upon Tyne: Sage.

[B14] Burke C. S. Stagl K. C. Salas E. Pierce L. Kendall D. (2006). Understanding team adaptation: a conceptual analysis and model. J. Appl. Psychol. 91, 1189–1207. 10.1037/0021-9010.91.6.1189 17100478

[B15] Carmeli A. Reiter-Palmon R. Ziv E. (2010). Inclusive leadership and employee involvement in creative tasks in the workplace: the mediating role of psychological safety. Creat. Res. J. 22, 250–260. 10.1080/10400419.2010.504654

[B16] Chan D. (1998). Functional relations among constructs in the same content domain at different levels of analysis: a typology of composition models. J. Appl. Psychol. 83, 234–246. 10.1037/0021-9010.83.2.234

[B17] Chen G. Kanfer R. (2006). Toward a systems theory of motivated behavior in work teams. Res. Org. Behav. 27, 223–267. 10.1016/S0191-3085(06)27006-0

[B18] Chen G. Kirkman B. L. Kanfer R. Allen D. Rosen B. (2007). A multilevel study of leadership, empowerment, and performance in teams. J. Appl. Psychol. 92, 331–346. 10.1037/0021-9010.92.2.331 17371082

[B19] Choi S. B. Tran T. B. H. Park B. I. (2015). Inclusive leadership and work engagement: mediating roles of affective organizational commitment and creativity. Soc. Behav. Personal. 43, 931–943. 10.2224/sbp.2015.43.6.931

[B20] Chowhan J. Pries F. Mann S. (2017). Persistent innovation and the role of human resource management practices, work organization, and strategy. J. Manag. Org. 23, 456–471. 10.1017/jmo.2016.8

[B21] Chun J. U. Yammarino F. J. Dionne S. D. Sosik J. J. Moon H. K. (2009). Leadership across hierarchical levels: multiple levels of management and multiple levels of analysis. Leadersh. Quart. 20, 689–707. 10.1016/j.leaqua.2009.06.003

[B22] Clegg C. Unsworth K. Epitropaki O. Parker G. (2002). Implicating trust in the innovation process. J. Occup. Org. Psychol. 75, 409–422. 10.1348/096317902321119574

[B23] Cohen S. G. Bailey D. E. (1997). What makes teams work: group effectiveness research from the shop floor to the executive suite. J. Manag. 23, 239–290. 10.1177/014920639702300303

[B24] Collins C. J. Smith K. G. (2006). Knowledge exchange and combination: the role of human resource practices in the performance of high-technology firms. Acad. Manag. J. 49, 544–560. 10.5465/amj.2006.21794671

[B25] Echols S. (2009). Transformational/servant leadership: a potential synergism for an inclusive leadership style. J. Relig. Leadersh. 8, 85–116.

[B26] Edmondson A. C. (1996). Learning from mistakes is easier said than done: group and organizational influences on the detection and correction of human error. J. Appl. Behav. Sci. 32, 5–28. 10.1177/0021886396321001

[B27] Edmondson A. C. (1999). Psychological safety and learning behavior in work teams. Admin. Sci. Quart. 44, 350–383. 10.2307/2666999

[B28] Edmondson A. C. (2004). Psychological safety, trust, and learning in organizations: a group level lens, in Trust and Distrust in Organizations: Dilemmas and Approaches, eds Kramer R. M. Cook K. S. (New York, NY: Russell Sage Foundation), 239–272.

[B29] Edmondson A. C. Lei Z. (2014). Psychological safety: the history, renaissance, and future of an interpersonal construct. Ann. Rev. Org. Psychol. Org. Behav. 1, 23–43. 10.1146/annurev-orgpsych-031413-091305

[B30] Edmondson A. C. Mogelof J. P. (2005). Explaining psychological safety in innovation teams, in Creativity and Innovation in Organizations, eds Thompson L. Choi H. (Mahwah, NJ: Erlbaum), 109–136.

[B31] Govendo J. A. (2005). Workforce, diversity and corporate creativity. Handb. Bus. Strategy 6, 213–218. 10.1108/08944310510557495

[B32] Hackman J. R. (2002). Leading Teams: Setting the Stage for Great Performance. Boston, MA: Harvard Business School Press.

[B33] Hair J. F. Anderson R. E. Tatham R. L. Black W. C. (1998). Multivariate Data Analysis, 5th Edn. Hoboken, NJ: Prentice Hall.

[B34] Harrington D. M. Block J. H. Block J. (1987). Testing aspects of Carl Rogers's theory of creative environments: child-rearing antecedents of creative potential in young adolescents. J. Personal. Soc. Psychol. 52, 851–856. 10.1037/0022-3514.52.4.851 3572740

[B35] Harrison D. A. Price K. H. Bell M. P. (1998). Beyond relational demography: time and the effects of surface- and deep-level diversity on work group cohesion. Acad. Manag. J. 41, 96–107. 10.5465/256901

[B36] Hirak R. Peng A. C. Carmeli A. Schaubroeck J. M. (2012). Linking leader inclusiveness to work unit performance: the importance of psychological safety and learning from failures. Leadersh. Quart. 23, 107–117. 10.1016/j.leaqua.2011.11.009

[B37] Hollander E. P. (2009). Inclusive Leadership: The Essential Leader-Follower Relationship. London: Routledge.

[B38] Hu J. Erdogan B. Jiang K. Bauer T. N. Liu S. (2018). Leader humility and team creativity: the role of team information sharing, psychological safety, and power distance. J. Appl. Psychol. 103, 313–323. 10.1037/apl0000277 29094959

[B39] Huang C. Chu C. Jiang P. (2008). An empirical study of psychological safety and performance in technology R&D teams, in Paper Presented at the 4th IEEE International Conference on Management of Innovation and Technology. Bangkok. 10.1109/ICMIT.2008.4654580

[B40] Hülsheger U. R. Anderson N. Salgado J. F. (2009). Team-level predictors of innovation at work: a comprehensive meta-analysis spanning three decades of research. J. Appl. Psychol. 94, 1128–1145. 10.1037/a0015978 19702361

[B41] James L. R. (1982). Aggregation bias in estimates of perceptual agreement. J. Appl. Psychol. 67, 219–229. 10.1037/0021-9010.67.2.219

[B42] James L. R. Demaree R. G. Wolf G. (1984). Estimating within-group interrater reliability with and without response bias. J. Appl. Psychol. 69, 85–98. 10.1037/0021-9010.69.1.85

[B43] James L. R. Demaree R. G. Wolf G. (1993). RWG: An assessment of within-group inter-rater agreement. J. Appl. Psychol. 78, 306–309. 10.1037/0021-9010.78.2.306

[B44] Janssen O. (2002). Transformational leadership and innovative work behavior of employees: a question of approachability of the leader. Behav. Org. 15, 275–293.

[B45] Janssen O. (2005). The joint impact of perceived influence and supervisor supportiveness on employee innovative behaviour. J. Occup. Org. Psychol. 78, 573–579. 10.1348/096317905X25823

[B46] Javed B. Abdullah I. Zaffar M. A. ul Haque A. Rubab U. (2019a). Inclusive leadership and innovative work behavior: the role of psychological empowerment. J. Manag. Org. 25, 554–571. 10.1017/jmo.2018.50

[B47] Javed B. Khan A. K. Quratulain S. (2018). Inclusive leadership and innovative work behavior: examination of LMX perspective in small capitalized textile firms. J. Psychol. 152, 594–612. 10.1080/00223980.2018.1489767 30260768

[B48] Javed B. Naqvi S. M. M. R. Khan A. K. Arjoon S. Tayyeb H. H. (2019b). Impact of inclusive leadership on innovative work behavior: the role of psychological safety. J. Manag. Org. 25, 117–136. 10.1017/jmo.2017.3

[B49] Kark R. Carmeli A. (2009). Alive and creating: the mediating role of vitality and aliveness in the relationship between psychological safety and creative work involvement. J. Org. Behav. 30, 785–804. 10.1002/job.571

[B50] Kelly E. Dobbin F. (1998). How affirmative action became diversity management: employer response to antidiscrimination law, 1961 to 1996. Am. Behav. Sci. 41, 960–984. 10.1177/0002764298041007008

[B51] Kozlowski S. W. Gully S. M. McHugh P. P. Salas E. Cannon-Bowers J. A. (1996). A dynamic theory of leadership and team effectiveness: developmental and task contingent leader roles. Res. Person. Hum. Resour. Manag. 14, 253–306.

[B52] Kozlowski S. W. J. Bell B. S. (2003). Work groups and teams in organizations, in Comprehensive Handbook of Psychology: Vol. 12. Industrial and Organizational Psychology, eds Borman W. C. Ilgen D. R. Klimoski R. J. (Hoboken, NJ: Wiley), 333–375.

[B53] Landis R. S. Beal D. J. Tesluk P. E. (2000). A comparison of approaches to forming composite measures in structural equation models. Org. Res. Methods 3, 186–207. 10.1177/109442810032003

[B54] Lovelace K. Shapiro D. L. Weingart L. R. (2001). Maximizing cross-functional new product teams' innovativeness and constraint adherence: a conflict communications perspective. Acad. Manag. J. 44, 779–793. 10.5465/3069415

[B55] Mathisen G. E. Einarsen S. Mykletun R. (2012). Creative leaders promote creative organizations. Int. J. Manpower 33, 367–382. 10.1108/01437721211243741

[B56] Mohammadi A. Broström A. Franzoni C. (2017). Workforce composition and innovation: how diversity in employees' ethnic and educational backgrounds facilitates firm-level innovativeness. J. Product Innov. Manag. 34, 406–426. 10.1111/jpim.12388

[B57] Mor Barak M. E. (2007). Managing diversity: toward a globally inclusive workplace. Acad. Manag. Learn. Educ. 6, 285–286. 10.5465/amle.2007.25223469

[B58] Mor Barak M. E. (2013). Managing Diversity: Toward a Globally Inclusive Workplace, 3rd Edn. Newcastle upon Tyne: SAGE.

[B59] Mor Barak M. E. Findler L. Wind L. H. (2003). Cross-cultural aspects of diversity and well-being in the workplace: an international perspective. J. Soc. Work Res. Eval. 4, 145–169.

[B60] Morgeson F. P. DeRue D. S. Karam E. P. (2010). Leadership in teams: a functional approach to understanding leadership structures and processes. J. Manag. 36, 5–39. 10.1177/0149206309347376

[B61] Nembhard I. M. Edmondson A. C. (2006). Making it safe: the effects of leader inclusiveness and professional status on psychological safety and improvement efforts in health care teams. J. Org. Behav. 27, 941–966. 10.1002/job.413

[B62] Nishii L. H. Rich R. E. (2014). Creating inclusive climates in diverse organizations, in Diversity at Work: The Practice of Inclusion, eds Ferdman B. M Deane B. R. (San Francisco, CA: Jossey-Bass), 205–228. 10.1002/9781118764282.ch11

[B63] Pelled L. H. Ledford G. E. Jr Mohrman S. A. (1999). Demographic dissimilarity and workplace inclusion. J. Manag. Stud. 36, 1013–1031. 10.1111/1467-6486.00168

[B64] Pless N. Maak T. (2004). Building an inclusive diversity culture: principles, processes and practice. J. Bus. Ethics 54, 129–147. 10.1007/s10551-004-9465-8

[B65] Podsakoff P. M. MacKenzie S. B. Lee J. Y. Podsakoff N. P. (2003). Common method biases in behavioral research: a critical review of the literature and recommended remedies. J. Appl. Psychol. 88, 879–903. 10.1037/0021-9010.88.5.879 14516251

[B66] Qi L. Liu B. (2017). Effects of inclusive leadership on employee voice behavior and team performance: the mediating role of caring ethical climate. Front. Commun. 2:8. 10.3389/fcomm.2017.00008

[B67] Randel A. E. Galvin B. M. Shore L. M. Ehrhart K. H. Chung B. G. Dean M. A.. (2018). Inclusive leadership: realizing positive outcomes through belongingness and being valued for uniqueness. Hum. Resour. Manag. Rev. 28, 190–203. 10.1016/j.hrmr.2017.07.002

[B68] Roberson Q. M. (2006). Disentangling the meanings of diversity and inclusion in organizations. Group Org. Manag. 1098, 212–236. 10.1177/1059601104273064

[B69] Schriesheim C. A. Castro S. L. Zhou X. DeChurch L. A. (2006). An investigation of path-goal and transformational leadership theory predictions at the individual level of analysis. Leadersh. Quart. 17, 21–38. 10.1016/j.leaqua.2005.10.008

[B70] Shore L. M. Randel A. E. Chung B. G. Dean M. A. Holcombe Ehrhart K. Singh G. (2011). Inclusion and diversity in work groups: a review and model for future research. J. Manag. 37, 1262–1289. 10.1177/014920631038594335213442

[B71] Siemsen E. Roth A. V. Balasubramanian S. Anand G. (2009). The influence of psychological safety and confidence in knowledge on employee knowledge sharing. Manufact. Service Operat. Manag. 11, 429–447. 10.1287/msom.1080.023323614632

[B72] Smith A. N. Morgan W. B. King E. B. Hebl M. R. Peddie C. I. (2012). The ins and outs of diversity management: the effect of authenticity on outsider perceptions and insider behaviors. J. Appl. Soc. Psychol. 42, 21–55. 10.1111/j.1559-1816.2012.01021.x

[B73] Sobel M. E. (1982). Asymptotic intervals for indirect effects in structural equations models, in Sociological Methodology, ed Leinhart S. (San Francisco, CA: Jossey-Bass), 290–312. 10.2307/270723

[B74] Tang N. Jiang Y. Chen C. Zhou Z. Chen C. C. Yu Z. (2015). Inclusion and inclusion management in the Chinese context: an exploratory study. Int. J. Hum. Resour. Manag. 26, 856–874. 10.1080/09585192.2014.985326

[B75] Tang N. Zhang K. (2015). Inclusive leadership: review and prospects. Chin. J. Manag. 12, 932–938. 10.3969/j.issn.1672-884x.2015.06.01935287502

[B76] Wang X. H. F. Howell J. M. (2012). A multilevel study of transformational leadership, identification, and follower outcomes. Leadersh. Quart. 23, 775–790. 10.1016/j.leaqua.2012.02.001

[B77] Wasserman I. C. Gallegos P. V. Ferdman B. M. (2008). Dancing with resistance: leadership challenges in fostering a culture of inclusion, in Diversity Resistance in Organizations, ed Thomas K. M. (Oxfordshire: Taylor and Francis), 175–200.

[B78] Welbourne T. M. Johnson D. E. Erez A. (1998). The role-based performance scale: validity analysis of a theory-based measure. Acad. Manag. J. 41, 540–555. 10.5465/256941

[B79] Winters M. (2014). From diversity to inclusion: an inclusion equation, in Diversity at Work: The Practice of Inclusion, eds Ferdman B. M. Deane B. R. (San Francisco, CA Jossey-Bass), 205–228. 10.1002/9781118764282.ch7

[B80] Wu W. (2008). Dimensions of social capital and firm competitiveness improvement: the mediating role of information sharing. J. Manag. Stud. 45, 122–146. 10.1111/j.1467-6486.2007.00741.x

[B81] Yammarino F. J. Dansereau F. (2008). Multi-level nature of and multi-level approaches to leadership. Leadersh. Quart. 19, 135–141. 10.1016/j.leaqua.2008.01.00125634038

[B82] Ye Q. Wang D. Guo W. (2019). Inclusive leadership and team innovation: the role of team voice and performance pressure. Eur. Manag. J. 37, 468–480. 10.1016/j.emj.2019.01.006

